# Talking welfare: the importance of a common language

**DOI:** 10.1007/s00335-015-9591-x

**Published:** 2015-08-19

**Authors:** James Bussell, Sara E. Wells

**Affiliations:** Welcome Trust Sanger Institute, Wellcome Trust Genome Campus, Hinxton, Cambridgeshire CB10 1SA UK; Mary Lyon Centre, MRC Harwell, Nineteenth St, Didcot, OXON OX11 0RD UK

## Abstract

Ontologies describing mouse phenotypes and pathology are well established and becoming more universally used (Smith and Eppig in Mamm Genome 23:653, 2012; Scofield et al. in J Biomed Semant 4:18, 2013). However, the language used to describe and disseminate cage-side observations is less well developed. This article explores the hurdles to unifying a language and terminology, and introduces our initial attempt to do so.

## Introduction

In 2013, in the United Kingdom alone, over 2 million genetically altered (GA) mice were bred for experimentation, an increase of 573 % from 1995 (UK Home Office; Annual Statistics of Scientific Procedures on Living Animals Great Britain 2013). This rise is a signal of a change in the populations of laboratory mice housed by many science support facilities. A decade or more ago, the range of genetically altered mice would have been much less; however, these individual colonies are likely to have been bigger in terms of mouse numbers and more homogenous in terms of genotype. The increased complexity of transgenesis and the growth in the number of genes in the mouse genome being manipulated has resulted in rapid rises in the number of genetically distinct colonies, many containing more than one altered allele. Concomitantly, through the refinement of experimentation and the advancement of molecular techniques such as genotyping and sequencing, the size of these colonies in terms of mouse numbers, has generally decreased. For example, it is now possible to genotype mice more rapidly at earlier ages, thereby ensuring that the only mice kept for any length of time are those of the required genotype. Multi-functional alleles such as those described by Skarnes et al. (2011) allow the generation of several allele types from a single targeting event. In addition, techniques such as qPCR, digital PCR and sequencing have enabled genome modifications such as deletions, translocations and copy counting of transgene insertions to be diagnosed molecularly instead of by costly mating methods.

This shift to more complex, diverse colonies poses a challenge in terms of assessing the wellbeing of the individual mice as different colonies present with different needs. Indeed, as we explore a wider range of genes and study the pleiotropic effects of genes (White et al. 2013), welfare issues become more unpredictable, necessitating strict regimes to be instigated. Cage-side observations of the abnormal behaviours and appearance of laboratory mice by animal care staff and researchers has proven vital for detection of phenotypes and more importantly for highlighting welfare concerns where the animal’s wellbeing is compromised. In the case of newly generated GA mouse lines, there is no doubt that characteristics and behaviours detected during routine husbandry practices such as cage changing can inform and direct research. However, these observations are sometimes subjective, recorded by non-research staff and susceptible to over-interpretation and even anthropomorphism. Indeed, many facilities have developed local cultures and languages to describe these events which aren’t recognised by other researchers or facilities.

### Welfare assessment

The guides to good animal welfare assessment (Hawkins et al. 2011; Wells et al. 2006) agree that frequent (at least daily) observations of laboratory animals by trained and competent staff should take place. Training of staff should include a thorough appreciation of the normal animal appearance and behaviour of the appropriate strains of mice. Such training should result in the animal care staff being able to detect subtle divergences from what is the expected coat conditions and key anatomical features such as shape and position of the ears, limb and tail appearance, shape and cutting edge of the teeth. Furthermore, staff must be able to recognise anomalies in movements, social interactions with other mice and general behaviour within the cage and when handled. Assessment criteria should evaluate the knowledge of the staff member and practical experience should be assured. Records of training and assessment should be maintained with periodic reassessment to ensure consistency. Such programmes have been highlighted as part of the EU directive 2010/63 and are explored as part of the working document on a common EU education and training framework (National Competent Authorities for the implementation of Directive 2010/63 2014).

The usefulness of the recorded observations relies not only on the description of a single observation but also the context in which the assessment takes place. Systems for recording welfare issues should make provision for and differentiate between the following:

- Reoccurring adverse effects: Phenotypes such as seizures, gait abnormalities and tremors which can be late onset, progressive and initially sporadic. This necessitates ensuring the entire welfare record of the mouse is kept and referred back to.
- Non-procedurally related harms: laboratory mice, even non-experimental mice with no genetic alteration will sometimes suffer from ill health. This can be a consequence of housing, infection or the underlying predisposition to some types of diseases that many laboratory wild-type mouse strains harbour (Szymanska et al. 2014).

### Welfare language

**Fig. 1:**
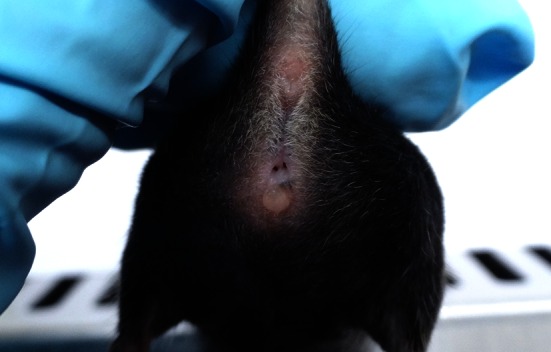
Vaginal Septum bifurcating the vulva and vagina of a C57BL/6J wild-type mouse
- *Objective and not interpreted* Many observable adverse effects can suggest a specific disease state or abnormal behaviour. However, without substantiating evidence, this cannot be inferred. For example, hair loss on a mouse may be because of barbering by cage mates or over grooming by the individual animal itself, but unless these behaviours are observed first hand, this should not be recorded as being the case.
- *Specific to a body location* The welfare observation should be hierarchical giving a standardised description of what you are observing and where. For example defining the gross region, the anatomical location and the observation allows the researcher, veterinarian or facility staff to firstly understand where the welfare concerns are impacting and secondly to associate the observation with similar trends in the same genetically altered strain of mice.
- *The inclusion of metadata* The age, husbandry conditions and experimental procedures undergone are amongst the key data required in order to make full use of welfare assessment.

With the globalisation of biomedical science has come the transfer of many thousands of different strains of mice around the world. Facilitated by many databases including the International Mouse Strain Resource—IMSR (http://www.findmice.org/), researchers have been able to import multiple strains for which there is a previous history of welfare assessment. Unfortunately the usefulness of this assessment is dependent upon how, why and by whom the information has been gathered. There are several guiding principles, however that we must strive to adhere to make sure that welfare terminology progresses to become a controlled language or ontology, understood by all and searchable by bioinformaticians:

- *A description not a diagnosis* Welfare assessment must describe what the animal care staff or researcher sees and not attempt to diagnose the underlying disease. For example, a swelling under the skin should be recorded as just that, not a tumour or an abscess which would require pathological interrogation.
- *Neither colloquial nor local definitions* The terminology used must be recognised by its veterinarian or biological descriptions, not locally applied terms. For example, an intact vaginal septum (Fig. 1, Gearhart et al. 2004) has in the past been described as ‘threading’ in one UK facility and ‘imperforate vagina’ in another. The local description was not understood in the other facility and would have undoubtedly caused confusion in others.

### Mouse welfare terms (http://www.mousewelfareterms.org)

**Fig. 2:**
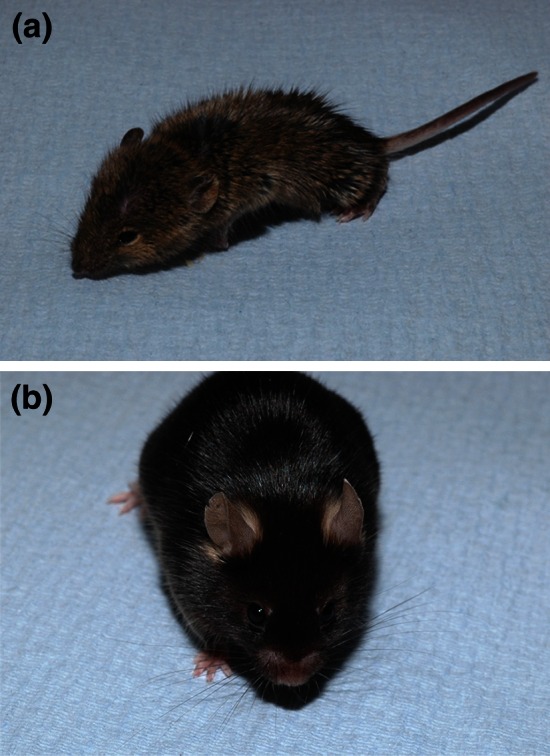
Mouse welfare terms are hierarchical and descriptive: **a** Appearance_whole body Coat/Skin_Coat Piloerection—Staring coat, bristling of hairs. **b** Head_ nose_shortened nose

We have produced a list of standardised descriptions for visible (behavioural and anatomical) characteristics affecting mouse welfare. It includes a hierarchical structure for describing the welfare that you wish to note and a standardised description of what you are observing, for example a mouse displaying a lump on its abdomen would be assigned the annotation *Abdomen_Coat/Skin_Swelling under the skin*. This mouse welfare terms list is compliant with the guiding principles above and has the potential to develop to link and integrate with mouse ontologies (Fig. 2).

### Reproducibility and records for the future

It should be recognised that the Mouse Welfare Terms are positioned to aid technical and scientific staff in communicating the observations they make in a clear and reproducible manner. Other initiatives such as the FELASA Working Group on Assessing Clinical Signs in Laboratory Animals (2015) aim to standardise clinical observations using veterinary terms. These efforts are complementary in enhancing clear communication to support animal welfare. The future will see the use of GA mouse strains increasing with global initiatives such as the International Mouse Phenotyping Consortium (www.mousephenotype.org) seeking to phenotype the protein coding genes within the mouse genome. The underlying driver for this group is the production and dissemination of standardised mouse models and data that can be used to inform human disease states. Reproducible data has allowed researchers to link phenotypic observations through the use of standard operating procedures shared amongst the contributing phenotyping centres. However, the observations that are made cage side can often go unreported if a clear mechanism for gathering such observations is not implemented. Inclusion of structured lists such as the mouse welfare terms allows animal care staff, veterinarians and research staff to communicate in a clear and reproducible manner. Greater adoption and development of standardised terms for the description and dissemination of welfare issues will lead to better exchange of information informing the scientific research and better supporting animal care.
